# Increase in Leptin and PPAR-γ Gene Expression in Lipedema Adipocytes Differentiated in vitro from Adipose-Derived Stem Cells

**DOI:** 10.3390/cells9020430

**Published:** 2020-02-12

**Authors:** Sara Al-Ghadban, Zaidmara T. Diaz, Hallie J. Singer, Karya B. Mert, Bruce A. Bunnell

**Affiliations:** 1 Center for Stem Cell Research and Regenerative Medicine, Tulane University, New Orleans, LA 70112, USA; zdiaz@tulane.edu (Z.T.D.); hsinger2@tulane.edu (H.J.S.); kmert@tulane.edu (K.B.M.); 2 Department of Pharmacology, Tulane University School of Medicine, New Orleans, LA 70112, USA

**Keywords:** lipedema, adipose-derived stem cells (ASCs), stromal vascular fraction (SVF) adipocyte differentiation, adipogenesis, inflammation

## Abstract

Lipedema is a painful loose connective tissue disorder characterized by a bilaterally symmetrical fat deposition in the lower extremities. The goal of this study was to characterize the adipose-derived stem cells (ASCs) of healthy and lipedema patients by the expression of stemness markers and the adipogenic and osteogenic differentiation potential. Forty patients, 20 healthy and 20 with lipedema, participated in this study. The stromal vascular fraction (SVF) was obtained from subcutaneous thigh (SVF-T) and abdomen (SVF-A) fat and plated for ASCs characterization. The data show a similar expression of mesenchymal markers, a significant increase in colonies (*p* < 0.05) and no change in the proliferation rate in ASCs isolated from the SVF-T or SVF-A of lipedema patients compared with healthy patients. The leptin gene expression was significantly increased in lipedema adipocytes differentiated from ASCs-T (*p* = 0.04) and the PPAR-γ expression was significantly increased in lipedema adipocytes differentiated from ASCs-A (*p* = 0.03) compared to the corresponding cells from healthy patients. No significant changes in the expression of genes associated with inflammation were detected in lipedema ASCs or differentiated adipocytes. These results suggest that lipedema ASCs isolated from SVF-T and SVF-A have a higher adipogenic differentiation potential compared to healthy ASCs.

## 1. Introduction

Lipedema is a painful loose connective tissue disorder that affects approximately 11% of adult women in the United States [1,2]. This disorder is characterized by a symmetrical increase of fat deposition in the legs and the arms, sparing the hands and the feet [3,4,5]. The symptoms of lipedema are often confused with obesity, lymphedema, chronic venous insufficiency and other fat disorders [6,7,8,9,10]. Lipedema consists of three stages characterized by the texture of skin and tissue formation: Stage 1 has normal smooth skin over pearl-sized nodules in a hypertrophic fat layer; Stage 2 has skin indentations over a hypertrophic fat structure of pearl to-apple-size masses; and Stage 3 has large extrusions of tissue causing skin and fat deformations mainly on the thighs and around the knees. [11,12,13,14]. Women with lipedema experience pain, heaviness of the affected limb, psychosocial distress, anxiety, eating disorders and the inability to lose weight as the fat tissue is highly resistant to diet and exercise [2,7,12,15,16,17]. Symptoms may progress in the advanced stages of lipedema and are associated with increased cardiovascular and renal diseases [1,2].

There is no cure for lipedema. Liposuction is by the far the most effective treatment to decrease the fibrotic lipedema fat and thereby maintain mobility, which is essential for the quality of life of women affected by lipedema [18,19,20,21]. Complex physical decongestive therapies (CPDT) including manual lymphatic drainage and compression with shock wave therapy (SWT) have been shown to improve the tissue structure and blood circulation [22,23]. A study conducted by Siems et al. showed that treating lipedema patients with CPDT and/or SWT reduced oxidative stress and cellulite, demonstrated by decreased levels of the lipid peroxidation marker (malondialdehyde) and protein carbonyls [24]. Although pain and bruising were still persistent, patients reported the effectiveness of these therapies. Furthermore, clinicians recommend supplements such as bioflavonoids, turmeric and selenium to lipedema patients, as these act as antioxidants which might help in reducing inflammation, improving blood vessels and removing free radicals [2].

While the etiology of lipedema remains unknown, hormones, genetic factors, inflammation, leaky and dilated blood and lymphatic vessels and the thickening of the interstitium are all known to contribute to the pathogenesis [12,25,26,27]. Our team and others have shown that lipedema adipose tissue is a highly vascularized and fibrotic tissue with an increase in blood vessels, infiltrating macrophages and hypertrophic adipocytes [25,28,29]. Suga et at. showed an increase in the proliferation of adipose-derived stem/progenitor/stromal cells (Ki67^+^ and CD34^+^ cells) in the adipose tissue of lipedema patients, indicating an increase in adipogenesis, leading to tissue hypoxia, adipocyte necrosis and macrophage infiltration [28].

Adding to that, Priglinger et al. [29] have characterized adipose-derived stromal/stem cells (ASCs) obtained from lipoaspirates and showed an increase in the number of CD146^+^ endothelial/pericytic cells in lipedema patients compared to healthy patients, proposing that this increase might be a marker of leaky blood and lymphatic vessels in lipedema adipose tissue. Thus, the expansion of adipose tissue, which is mediated by the increase in adipocyte size (hypertrophy) and/or the proliferation (hyperplasia) and differentiation (adipogenesis) of adipose progenitor cells/pre-adipocytes to mature adipocytes is detected in lipedema adipose tissue. Adipogenesis is a complex cellular differentiation process tightly regulated by key transcription factors such as peroxisome proliferator-activated receptor-γ (PPAR-γ), which in turn controls the expression of several other genes involved in lipogenesis (Lipoprotein lipase, LPL), insulin sensitivity (Glucose transporter type 4, GLUT4) and hormones such as leptin [30,31,32].

As lipedema primarily manifests in the legs of patients in early stages but extends to other areas in the body as the disease progresses, we characterized ASCs obtained from the subcutaneous adipose tissue of both the thigh (SVF-T) and abdomen (SVF-A) of lipedema patients and healthy controls. A full phenotypic characterization of ASCs as defined by the International Federation for Adipose Therapeutics and Science (IFATS) and the International Society for Cellular Therapy (ISCT) was performed, as was an evaluation of the gene expression of adipogenic and inflammatory markers in ASCs and differentiated adipocytes. Characterizing ASCs from different fat depots will provide insights into the biology of adipose fat tissue in lipedema patients and will provide a better understanding of their potential application in cellular therapy and regenerative medicine.

## 2. Materials and Methods

### 2.1. Participants

A total of forty patients, 20 with lipedema and 20 healthy controls (non-lipedema subjects) participated in this study. Lipoaspirates were harvested from thigh and abdomen obtained from women undergoing elective liposuction. For lipedema patients, both tissues are considered affected as diagnosed by the physicians performing the liposuctions. All subjects provided their informed consent for inclusion before they participated in the study. The study was conducted in accordance with the Declaration of Helsinki and the protocols were approved by the Human Research and Protection Program at the University of Arizona (Institutional Review Board (IRB) protocol number: 1602399502) and Tulane University (IRB protocol number: 9189). Participants were determined to have lipedema based on the criteria of Wold et al [4]. All patients’ identifiers were kept confidential and all received samples utilized in this study were de-identified. Table 1 provides the characteristics of the participants. No statistical significance in the age or Body Mass Index (BMI) was observed between the two groups.

### 2.2. ASCs Isolation and Cell Culture

ASCs were isolated from lipoaspirates of subcutaneous adipose tissue from the abdomen or thigh. The lipoaspirates were washed with 1Xphosphate-buffered saline (PBS) and incubated at 37 °C in a rocking incubator at 100 rpm for 1 h in 0.1% collagenase type 1 (Sigma, St. Louis, MO, USA). The digested tissue was then centrifuged to remove lipids, primary adipocytes and collagenase solution. The SVF pellet containing ASCs was plated in 175 cm^2^ flasks. ASCs were maintained in Dulbecco’s modified eagles medium (DMEM)/F12 (Hyclone, Logan, UT, USA) with 10% fetal bovine serum (FBS, Hyclone, Logan, UT, USA) and 1% antibiotic/antimycotic (Thermo Fisher Scientific, Waltham, MA, USA). Media was changed every three days until the cells achieved 80%–90% confluence. ASCs were culture expanded in the same medium and harvested at passage two (p2) with 0.25% trypsin/1 mM EDTA (Thermo Fisher Scientific, Waltham, MA, USA) and used for the experiments. ASCs from lipedema and non-lipedema participants were used at p3, plated at the same cell density and performed for the same duration of time for all the experiments.

The healthy ASCs-T used in this study were a generous gift from Dr. Eleni Priglinger (Ludwig Boltzmann Institute for Experimental and Clinical Traumatology, AUVA Research Center, Vienna, Austria).

### 2.3. Alamar Blue Cell Proliferation Assay

ASCs were seeded at a density of 15.6 × 10^3^ cells/cm^2^ in a 96-well plate. The cell proliferation was assessed on days 1, 7, 14 and 21 in culture. The cells were incubated with 20 μL of AlamarBlue reagent (Thermo Fisher Scientific, Waltham, MA, USA) for 2 h in an incubator at 37 °C with 5% CO_2_. The fluorescence intensity was measured with excitation at 540 nm and emission at 600 nm on a Synergy™ HTX Multi-Mode Microplate Reader (BioTek, Winooski, VT, USA).

### 2.4. Colony-Forming Unit (CFU) Fibroblast Assay

ASCs were seeded at a density of 0.5 × 10^2^ cells/cm^2^ in a 6-well plate and were cultured for 14 days. The medium was changed on day seven after cell seeding. At day 14, the cells were washed twice with 1XPBS and stained with 1 mL of 3% crystal violet (Sigma, St. Louis, MO, USA) for 30 min at room temperature (RT). The plates were then washed with deionized water and placed on the rocker for 10 min. The number of colonies was manually quantified, with only CFUs greater than 2 mm^2^ in diameter being recorded. Each experiment was performed in triplicate.

### 2.5. Flow Cytometry

For the phenotypic analysis, the cells were blocked with 1% BSA and 1% CD16/CD32 in 1XPBS and stained with the following antibodies at RT for 15 min: CD3 (Cat #: 562406, BD Biosciences, San Jose, CA, USA), CD14 (Cat #: IM2640U, Beckman-Coulter, USA), CD31 (Cat #: 563651, BD Biosciences, USA), CD45 (Cat #: A71117, Beckman-Coulter Brea, CA, USA), CD73 (Cat #: 550257, BD Biosciences, USA), CD90 (Cat #: 11-0909-42, Invitrogen, Waltham, MA, USA) and CD105 (Cat #: 17-1057-42, Invitrogen, USA). The cells were then fixed with 1% paraformaldehyde (PFA) and a total of 10,000 events were captured and analyzed with a Gallios Flow Cytometer using Kaluza software (Beckman Coulter, Brea, CA, USA).

### 2.6. Adipogenic Differentiation and Oil Red O Staining

For the adipogenic differentiation, ASCs were seeded at a density of 2 × 10^4^ cells/cm^2^ and grown in DMEM/F12. At confluence, the medium was replaced with adipogenic differentiation-inducing medium (AdipoQual, LaCell, NO, LA, USA) for the differentiation of the cells, or kept in DMEM/F12 medium for control undifferentiated cells. Media was changed every three days until day 21. For staining, the control and differentiated cells were fixed with 4% PFA for 30 min, stained with filtered Oil Red O (Sigma, St. Louis, MO, USA) for 15 min, followed by multiple rinses with 1XPBS. These cells were then visualized using a Nikon Eclipse TE200 with Nikon Digital Camera DXM1200F and Nikon ACT-1 software version 2.7 at 10× and 20× magnification (Nikon, Melville, NY, USA). The absorbance of the Oil Red O eluted by adding 100% isopropanol was measured at 584 nm wavelength by spectrophotometry. The differentiation values are reported as a percent of the undifferentiated, control cells. For the transcriptional analysis, plates were washed twice with 1xPBS and stored at −80 °C for RNA extraction.

### 2.7. Osteogenic Differentiation and Alizarin Red Staining

For the osteogenic differentiation, the cells were seeded at a density of 2 × 10^4^ cells/cm^2^ and grown to confluence in DMEM/F12. Upon reaching confluency, DMEM/F12 media was replaced with osteogenic differentiation medium DMEM/F12 containing 10% FBS, 10 nM dexamethasone (Sigma, St. Louis, MO, USA), 50 µM ascorbic acid-2-phosphate (Sigma, USA) and 20 mM β-glycerophosphate (STEMCELL Technologies, Cambridge, MA, USA). The control cultures were cultured in DMEM/F12 medium. After 28 days, the osteogenic differentiation was analyzed with alizarin red staining, followed by extraction and quantification. For the alizarin red staining of the calcified structures, the cells were fixed in 4% PFA for 30 min at RT and stained with alizarin red solution. For the quantitative analysis of alizarin red staining, the cells were washed with deionized water and incubated with 10% cetylpyridinium chloride (CPC, Sigma, St. Louis, MO, USA) for 1 h. The absorbance was measured at 562 nm wavelength by spectrophotometry.

### 2.8. RNA Isolation and Quantitative Real-Time PCR (qRT-PCR)

The total RNA from undifferentiated ASCs and from induced-ASCs was extracted using an RNA extraction kit (Qiagen, Germantown, MD, USA) and then digested with DNase I (Qiagen, USA). A total of 1 µg of mRNA was used for the cDNA synthesis Applied Bioscience purification kit (Thermo Fisher Scientific, USA). qRT-PCR was performed using the SYBR Green qPCR SuperMix (Bio-Rad, Hercules, CA, USA), according to the manufacturer’s instructions. Oligonucleotide primers were designed with the vendor’s software (IDT, Coralville, IA, USA). Table 2 lists the primer sequences used for qRT-PCR. The PCR conditions were: 2 min at 95 °C and 40 cycles of 15 s at 95 °C and 30 s at 60 °C. The target and reference genes were amplified in separate wells. All reactions were performed in duplicate. The 2^−ΔΔCt^ method was used to calculate the relative fold change in the gene expression after normalization to GAPDH, which was used as an internal control.

### 2.9. Statistical Analysis

GraphPad PRISM 8 was used for all statistical analyses. A Mann-Whitney test was used to determine the differences between the two groups of participants. A one-way ANOVA followed by a Tukey’s post hoc test was used to analyze the differences between the four groups. Asterisks (*) indicate statistical significance: * *p* < 0.05; ** *p* < 0.01.

## 3. Results

### 3.1. Lipedema ASCs Isolated from SVF-T and SVF-A Showed a Significant Increase in CFU with no Change in Stemness Makers or Proliferation Rate Compared to Healthy ASCs

**Figure 1:**
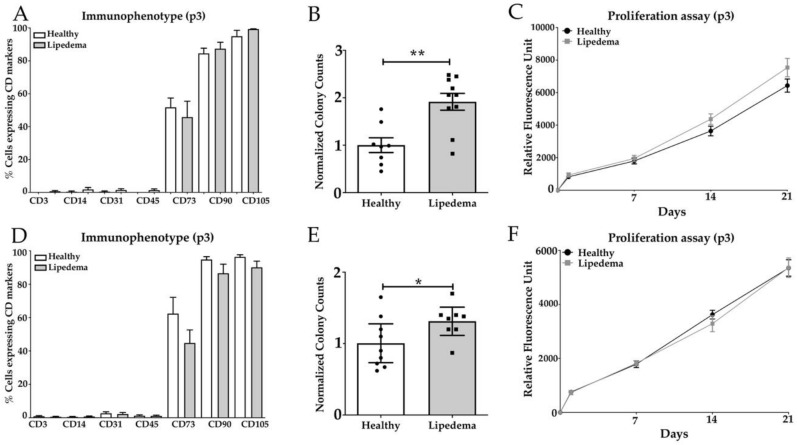
Characterization of (**A**–**C**) ASCs-T and (**D**–**F**) ASCs-A isolated from SVF-T and SVF-A of healthy and lipedema patients. (**A**,**D**) Flow cytometry analysis of the surface marker expression was similar for cultured ASCs between healthy and lipedema patients (*n* = 10 per group). (**B**,**E**) The quantitative analysis of the CFU assay revealed a significant increase in the colony-forming potential of ASCs from lipedema patients compared with healthy patients (ASCs-T: healthy *n* = 8 and lipedema *n* = 10; ASCs-A: healthy *n* = 9 and lipedema *n* = 8). The results are displayed as scatter plots with bars. The values are the mean ± SEM. * *p* < 0.05; ** *p* < 0.01. (**C**,**F**) The AlamarBlue assay showed no difference in the proliferation rate of ASCs between healthy and lipedema patients at 1, 7, 14 and 21 days in culture (*n* = 10 per group).

The phenotypic characterization of ASCs was analyzed by flow cytometry for the expression of stemness and surface markers. ASCs isolated from lipedema SVF-T and SVF-A showed a similar expression of mesenchymal stem cell (MSC) markers: CD73, CD90, CD105 to ASCs derived from SVF of healthy patients. CD90 and CD105 were highly expressed in ASC-T and ASC-A (90%–100%) isolated from the two cohort groups; CD73 was expressed in 50% of ASCs-T, but it ranged from 40% (lipedema) to 60% (healthy) in ASCs-A (Figure 1A,D). Other surface markers were expressed at a very low percentage in ASCs-T and ASCs-A of the two groups and they include the T-cell marker (CD3), monocyte/macrophage marker (CD14), endothelial marker (CD31) and the lymphohematopoietic marker (CD45) in ASC-T and ASC-A isolated from both lipedema and healthy patients (Figure 1A,D). Furthermore, the self-renewal potential of ASCs was measured by their ability to form fibroblast-like colonies. The quantitative analysis of the CFU showed a significant increase in the colony-forming potential of ASCs-T (*p* < 0.01) and ASCs-A (*p* < 0.05) derived from lipedema patients compared to healthy patients (Figure 1B,E). The cell proliferation was also assessed and the data showed no significant difference in the growth rate between lipedema ASCs-T and ASCs-A as compared to healthy ASCs after 1, 7, 14 and 21 days in culture (Figure 1C,F).

### 3.2. Lipedema ASCs Showed Increased Adipogenic but not Osteogenic Differentiation Potential as Compared to Healthy ASCs

**Figure 2:**
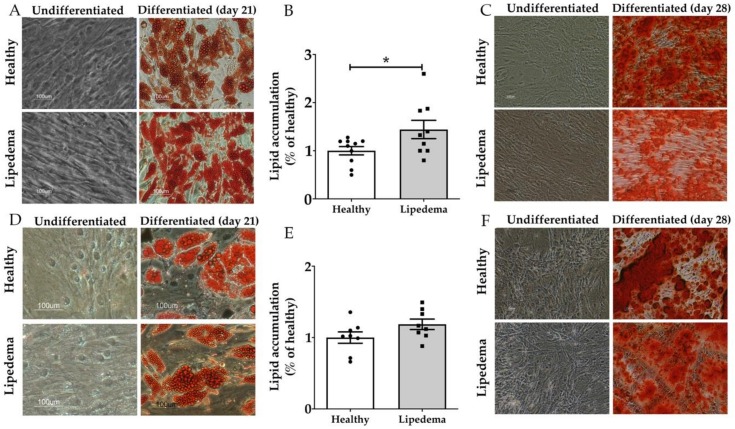
The in vitro adipogenic and osteogenic differentiation potential of (**A**–**C**) ASCs-T and (**D**–**F**) ASCs-A. (**A**,**D**) Representative photomicrographs of the adipogenic differentiation of ASCs determined by Oil Red O staining three weeks after the adipogenic induction. Lipid droplets (red) observed inside differentiated cells. No stain in the undifferentiated cells (scale bar, 100 µm). (**B**,**E**) The quantitative analysis of Oil Red O stain by spectrophotometric analysis showing a significant increase in the adipogenic differentiation potential of lipedema ASCs-T compared to healthy ASCs-T (ASCs-T: healthy *n* = 10 and lipedema *n* = 9, ASCs-A: *n* = 8 per group). The results are displayed as scatter plots with bars. The values are the mean ± SEM. * *p* < 0.05. (**C**,**F**) Representative photomicrographs of the osteogenic differentiation of ASCs determined by alizarin red staining four weeks after osteogenic induction (scale bar, 100 µm).

ASCs isolated from SVF-T and SVF-A of healthy and lipedema patients were differentiated into adipocytes, as shown by Oil Red O staining (Figure 2A,D) and osteoblasts as determined by alizarin red staining (Figure 2C,F). The quantitative analysis of Oil Red O staining showed a higher adipogenic differentiation potential of lipedema ASCs compared to healthy ASCs (Figure 2B,C), with a significant increase in lipedema ASCs-T (Figure 2B, *p* < 0.05). No difference was detected in osteogenic differentiation between the two groups, as confirmed by quantitative analysis (Figure S1).

### 3.3. Significant Increase in Leptin and PPAR-γ Gene Expression in Lipedema Differentiated Adipocytes Compared to Healthy Adipocytes

**Figure 3:**
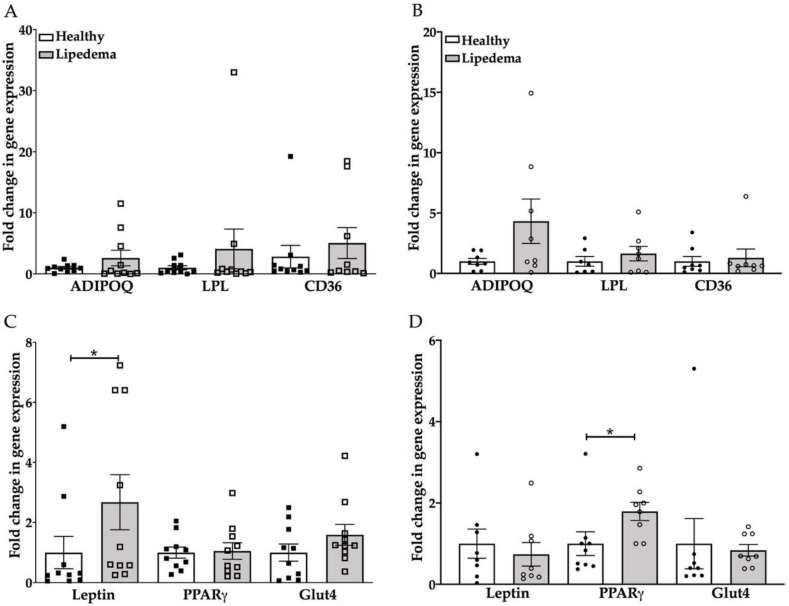
The adipogenic differentiation marker genes increased in differentiated adipocytes of lipedema (**A**,**C**) ASCs-T and (**B**,**D**) ASCs-A as compared to corresponding cells from healthy ASCs. (**A**,**B**) The qRT-PCR analysis showed a trend of increase in adiponectin, LPL and CD36 gene expression in differentiated adipocytes of lipedema ASCs-T and ASCs-A as compared to healthy differentiated adipocytes (ASCs-T: *n* = 10 per group; ASCs-A: *n* = 8 per group). (**C**,**D** ) The qRT-PCR analysis showed (**C**) a significant increase in leptin gene expression in differentiated adipocytes of lipedema ASCs-T and (**D**) a significant increase in PPAR-γ gene expression in differentiated adipocytes of lipedema ASCs-A, with no change in the Glut4 gene expression as compared to healthy differentiated adipocytes (ASCs-T: *n* = 10 per group; ASCs-A: *n* = 8 per group). The gene expression of differentiated adipocytes are calculated from the percent of undifferentiated, control cells. The results are displayed as scatter plots with bars. The values are the mean ± SEM. * *p* < 0.05. Abbreviations: ADIPOQ: adiponectin; LPL: Lipoprotein lipase; PPRA-γ: peroxisome proliferator-activated receptor gamma; and CD36: cluster of differentiation 36.

Adipogenesis of ASCs was further confirmed by qRT-PCR for adipogenic markers: adiponectin, LPL, CD36, Leptin, PPAR-γ and Glut4. Adipocytes differentiated from lipedema ASCs-T and ASCs-A showed an increase in adiponectin, LPL and CD36 gene expression compared to the same cells from healthy ASCs (Figure 3A,B). The increase was statistically insignificant due to the high variability observed in the samples. The leptin and PPAR-γ gene expression showed a significant increase in differentiated adipocytes from lipedema ASCs-T (*p* = 0.04) and ASCs-A (*p* = 0.03), respectively, compared to adipocytes differentiated from Healthy ASCs. The Glut4 gene expression was similar in the differentiated adipocytes of both groups (Figure 3C,D).

### 3.4. Inflammatory Genes Expression Was Similar for Lipedema ASCs and Adipocytes Compared to Healthy Cells

**Figure 4:**
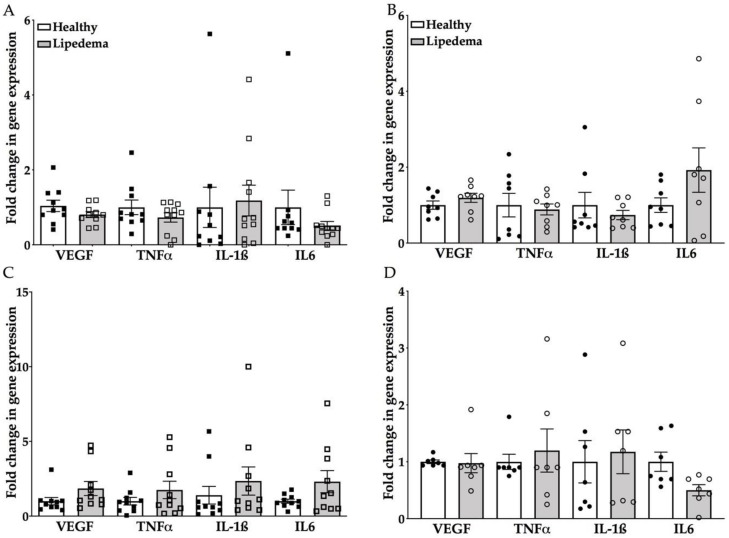
Inflammatory marker genes show a similar expression in (**A**,**B**) ASCs and (**C**,**D**) differentiated adipocytes between lipedema and healthy patients. (**A**,**B**) The qRT-PCR analyses of VEGF, IL-6, IL-1β and TNFα gene expression in (**A**) ASCs-T and (**B**) ASCs-A are similar when compared to healthy ASCs (ASCs-T: *n* = 10 per group; ASCs-A: *n* = 8 per group). (**C**,**D**) The qRT-PCR analysis showed (**C**) a trend of increase in all inflammatory markers in the differentiated adipocytes of lipedema ASCs-T (**D**) with no change in the differentiated adipocytes of lipedema ASCs-A (ASCs-T: *n* = 10 per group; ASCs-A: *n* = 8 per group). The gene expression of the differentiated adipocytes are calculated from the percent of undifferentiated, control cells. The results are displayed as scatter plots with bars. The values are the mean ± SEM.

As obesity induces adipose tissue remodeling, fibrosis and angiogenesis, these changes alter ASCs’ immune phenotype and function, as demonstrated by the increased expression of pro-inflammatory cytokines, such as interleukin 1 beta (IL-1β), IL-6, tumor necrosis factor-alpha (TNF-α) and vascular endothelial growth factor (VEGF) [33,34]. Thus, the expression level of these genes was determined in lipedema and healthy ASCs by qRT-PCR. No difference was detected in the gene expression between ASCs-T and ASCs-A isolated from lipedema and healthy patients (Figure 4A,B). However, there was a trend of increase in the gene expression of VEGF, IL-6, IL-1β and TNFα in adipocytes differentiated from lipedema ASCs-T compared to the same cells differentiated from healthy patients (Figure 4C), but none was detected in adipocytes differentiated from lipedema ASCs-A compared to healthy cells (Figure 4D).

## 4. Discussion

As lipedema is a chronic, painful fat disorder characterized by a significant expansion of adipose tissue in the lower extremities of the body [5,12], ASCs residing in the adipose niche and stimulated by a plethora of inflammatory and angiogenic factors might contribute to tissue adiposity. ASCs are a heterogeneous population of cells characterized by their ability to adhere to plastic, proliferate in culture, form colonies and differentiate into multiple lineages in vitro and in vivo [35,36,37,38].

In this study, ASCs isolated from the SVF-T and SVF-A of 20 lipedema patients and 20 healthy subjects were characterized for their stemness markers, self-renewal ability and differentiation ability to adipocytes and osteoblasts in vitro. The phenotypic characterization of the SVF-T and SVF-A isolated from lipedema patients revealed the differential expression of stemness and surface markers. However, the differences were not statistically significant (Figure S2A). Interestingly, a significant increase in CD31, a vascular endothelial marker, was detected in SVF-T and SVF-A isolated from lipedema patients as compared to SVF-A from healthy patients (Figure S2B). Consistent with this, Priglinger et al. showed a significant increase in the endothelial/pericytic marker CD146 in SVF isolated from the hips and thigh of lipedema patients compared to healthy patients, proposing that this increase might be a marker of repair of leaky blood and lymphatic vessels in lipedema tissues [29].

Priglinger et al. also showed no difference in the MSC markers CD90, CD73 and CD105, as well as in the proliferation rate of ASCs isolated from SVF of lipedema patients compared to healthy patients [29], which is consistent with our findings on lipedema ASCs isolated from SVF-T but which contradicts the data published by Bauer et al. showing a significant increase in proliferation in lipedema ASCs at day 14 compared to non-lipedema ASCs [39]. Furthermore and in contrast to the data published by Priglinger et al. and Bauer et al., we showed a significant increase in the adipogenic potential of ASCs-T of lipedema patients compared with the same cells from healthy patients. These differences might be due to several factors, including different techniques used to perform the proliferation and differentiation assays, the use of different liposuction techniques, the harvest site and the disease stage of the patients enrolled in the study.

Additionally, we have isolated ASCs from SVF-A obtained from lipedema patients and showed that there is no difference in the expression of MSC markers or the proliferation rate compared with the same cells from healthy patients. Interestingly, lipedema ASCs-A displayed a slower proliferation rate when compared to lipedema ASCs-T, with no difference in their colony forming unit (Figure S3). Nevertheless, there was a significant decrease in the adipogenic and osteogenic differentiation potential of lipedema ASCs-A compared to lipedema ASCs-T (*p* < 0.05), as shown by the quantitative analysis in Figure S4. There was also a significant decrease in the osteogenic potential in healthy ASCs-A compared to healthy ASCs-T, but no difference was shown in their adipogenic potential (Figure S4A,B). Taken together, these results demonstrate that lipedema and healthy ASCs isolated from two diverse depots of adipose tissue display a distinctive clonogenic and differentiation potential toward adipogenic and osteogenic lineages, confirming the cellular specificity of ASCs, as previously in reported several studies [40,41,42].

Adipogenesis is a complex process of cellular differentiation of ASCs that has been widely used to study metabolic disorders and regenerative medicine [32,43,44,45]. In vitro, PPAR-γ is a key regulator of adipogenesis and its expression determines the commitment of preadipocytes’ differentiation into mature adipocytes [30,46]. In this study, we showed an increase in the adipogenic differentiation potential of lipedema ASCs isolated from SVF-T and SVF-A, as assessed by the degree of lipid droplet accumulation by Oil Red O stain and the expression of the main adipogenic gene markers: adiponectin, LPL, CD36, Leptin, PPAR-γ and Glut4. The PPAR-γ gene expression showed a significant increase in differentiated adipocytes from lipedema ASCs-A, but not from ASCs-T, compared to adipocytes differentiated from healthy ASCs. Although the PPAR-γ activity has been demonstrated to regulate insulin sensitivity [32,47], no change in the GLUT4 gene expression was detected in adipocytes differentiated from lipedema ASCs as compared to healthy ASCs.

Leptin is known to regulate energy homeostasis by controlling satiety and body weight and has been shown to be highly expressed by adipocytes differentiated from obese ASCs [48,49,50]. Our data showed a significant increase in differentiated adipocytes from lipedema ASCs-T compared to adipocytes differentiated from healthy ASCs. Comparing the adipogenic gene expression in differentiated adipocytes between the four groups resulted in no statistical difference among the lipedema or healthy groups (data not shown).

Lipedema adipose tissue is highly infiltrated with immune cells instigating an increase in fibrosis and angiogenesis. We and others have shown increased levels of macrophages observed around blood vessels or forming crown-like structures surrounding necrotic adipocytes [25,28,51]. Therefore, we ought to determine the expression levels of inflammatory genes in lipedema ASCs derived from SVF-T and SVF-A. Our results showed no difference in gene expression between ASCs-T and ASCs-A isolated from lipedema and healthy patients. However, there was a trend of increase in the gene expression of VEGF, IL-6, IL-1β and TNFα in adipocytes differentiated from lipedema ASCs-T, which can be correlated with the data we have previously published of a significant increase in macrophages detected by CD68 expression in lipedema adipose thigh fat tissue [25]. Additionally, the increase in VEGF transcription can also be related to previously reported data on elevated levels in the blood of ten women with lipedema compared to controls [24].

Having established the stemness of lipedema ASCs, our next step will be conducting experiments to investigate the usefulness of ASCs in developing a scaffold for tissue engineering and the potential treatment of the disease. However, one of the main limitations of this study is the scarcity of samples from lipedema patients with stage 1 and stage 3, as well as from lean healthy participants. The ability to conduct studies on lipedema stage 1 patients will provide insights on the early manifestation of the disease and will probably provide clues on how to prevent its progression.

## 5. Conclusions

In conclusion, we demonstrated that lipedema ASCs isolated from SVF-T show a higher adipogenic differentiation potential compared to lipedema ASCs isolated from SFV-A and healthy ASCs. Although ASCs might induce adipogenesis in lipedema, an in-depth characterization of ASCs will provide a better understating of the pathophysiology of lipedema and probably be a promising tool for lipedema patients.

## Figures and Tables

**Table 1:** Characteristics of healthy and lipedema patients.

Characteristics	Healthy	Lipedema
Number	20	20
Sex (M/F)	Female	Female
Age (years)	46.9 ± 2.2	44.1 ± 2.2
BMI (kg/m^2^)	28.5 ± 0.86	29.9 ± 0.97
Stage 1	−	15%
Stage 2		70%
Stage 2–3	−	5%
Stage 3	−	10%

**Table 2:** List of primers used for real-time quantitative PCR (qRT-PCR).

Name	Forward	Reverse
IL-6	5′-GTAGCCGCCCCACACAGACAGCC-3′	5′-GCCATCTTTGGAAGGTTC-3′
IL-1β	5′-TCCCCAGCCCTTTTGTTGA-3′	5′-TTAGAACCAAATGTGGCCGTG-3′
TNF-α	5′-TCTTCTCGAACCCCGAGTGA-3′	5′-CCTCTGATGGCACCACCAG-3′
VEGF	5′-CCTTGCTGCTCTACCTCCAC-3′	5′-CACACAGGATGGCTTGAAGA-3′
Glut4	5′-AGC AGC TCT CTG GCA TCA AT-3′	5′-CAA TGG AGA CGT AGC ACA TG-3′
CD36	5′-GAGACCTGCTTATCCAGAAGACAAT-3′	5′-TTCTGTGCCTGTTTTAACCCAATTTTT-3′
LPL	5′-GAGATTTCTCTGTATGGCACTG-3′	5′-CTGCAAATGAGACACTTTCTC-3′
Leptin	5′-GAAGACCACATCCACACACG-3′	5′-AGCTCAGCCAGACCCATCTA-3′
PPAR-γ	5′-AGGCGAGGGCGATCTTG-3′	5′-CCCATCATTAAGGAATTCATGTCATA-3′
ADIPOQ	5′-AACATGCCCATTCGCTTTAC-3′	5′-AGAGGCTGACCTTCACATCC-3′
GAPDH	5′-CGCTGAGTACGTCGTGGAGTC-3′	5′-GCAGGAGGCATTGCAGATGA-3′

## Supplementary Materials

The following are available online at https://www.mdpi.com/2073-4409/9/2/430/s1: Figure S1: Quantitative analysis of alizarin red by spectrophotometry showing no difference in the osteogenic differentiation between healthy and lipedema ASCs-T or healthy and lipedema ASCs-A (*n* = 4 per group). The results are displayed as scatter plots with bars. The values are the mean ± SEM; Figure S2: Flow cytometry analysis of SVFs. (A) SVFs showed a similar expression of stemness and surface markers between lipedema patients (SVF-A, *n* = 6; SVF-T, *n* = 8). (B) CD31 marker expression in SVFs showed a significant increase in lipedema patients compared to healthy patients (SVF-A healthy, *n* = 8; SVF-T lipedema, *n* = 6; SVF-A lipedema, *n* = 9). The results are displayed as scatter plots with bars. The values are the mean ± SEM. ** *p* < 0.01 *** *p* < 0.001; Figure S3: Quantitative analysis of CFU assay revealed a significant increase in the colony-forming potential of lipedema ASCs compared with healthy patients as well as a significant decrease between healthy ASCs-T and lipedema ASCs-A (ASCs-T: healthy *n* = 8 and lipedema *n* = 10; ASCs-A: healthy *n* = 9 and lipedema *n* = 8). Results are displayed as scatter plots with bar. Values are mean ± SEM. * *p* < 0.05, ** *p* < 0.01; Figure S4: Quantitative analysis of Oil Red O (A) and Alizarin red (B) by spectrophotometry showing a significant decrease in adipogenic and osteogenic differentiation potential in lipedema ASCs-A compared to lipedema ASCs-T (*p* < 0.05) and a significant decrease in the osteogenic potential in healthy ASCs-A compared to healthy ASCs-T, but no difference was shown in their adipogenic potential (Adipogenic differentiation ASCs-T: healthy *n* = 10 and lipedema *n* = 9, ASCs-A: *n* = 8 per group, Osteogenic differentiation, *n* = 4 per group).
