# Supplementary material for: Increase in Leptin and PPAR-γ Gene Expression in Lipedema Adipocytes Differentiated in vitro from Adipose-Derived Stem Cells
Source: Cells. 2020 Feb 12;9(2):430. doi: 10.3390/cells9020430 (PMC7072543; doi:10.3390/cells9020430)
Supplement: Supplementary file 1 [file cells-09-00430-s001.pdf]

cells MDPI

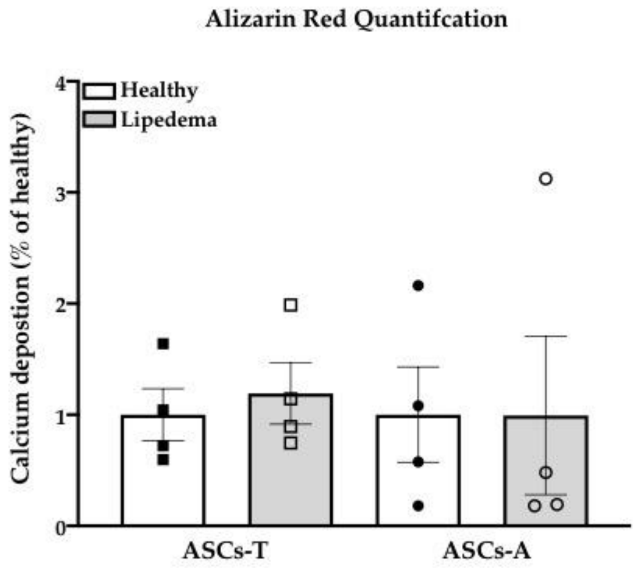

Figure S1. Quantitative analysis of alizarin red by spectrophotometry showing no difference in osteogenic differentiation between healthy and lipedema ASCs-T or healthy and lipedema ASCs-A (n= 4 per group). Results are displayed as scatter plots with bar. Values are mean ± SEM.

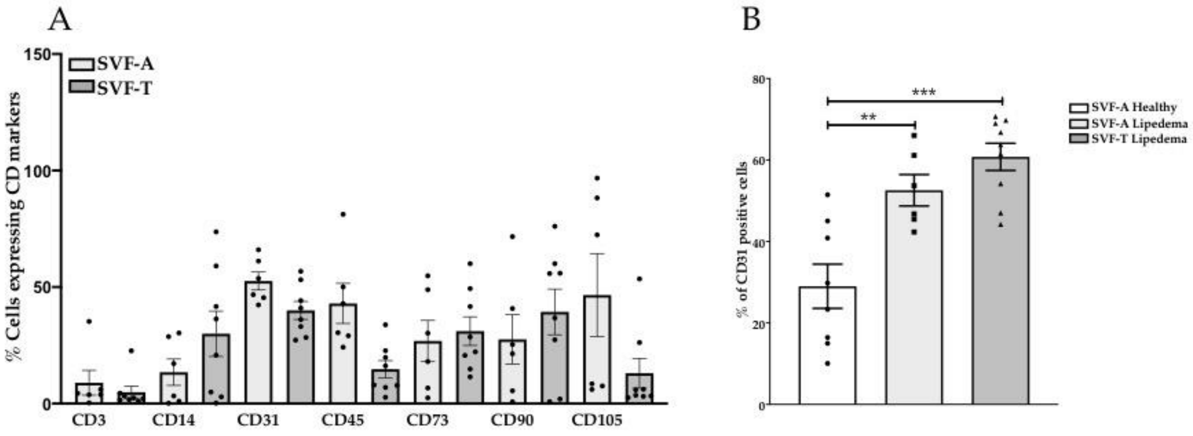

Figure S2. Flow cytometry analysis of SVFs. (A) SVFs showed a similar expression of stemness and surface markers between lipedema patients (SVF-A, n = 6; SVF-T, n = 8). (B) CD31 marker expression in SVFs showed a significant increase in lipedema patients compared to healthy patients (SVF-A healthy, n = 8; SVF-T lipedema, n = 6; SVF-A lipedema, n = 9). Results are displayed as scatter plots with bar. Values are mean ± SEM. ** $p < 0.01$ *** $p < 0.001$.

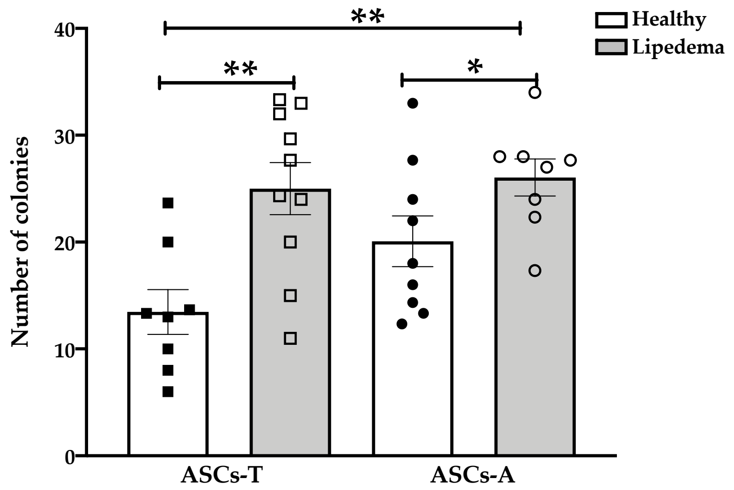

Figure S3. Quantitative analysis of CFU assay revealed a significant increase in the colony-forming potential of lipedema ASCs compared with healthy patients as well as a significant decrease between healthy ASCs-T and lipedema ASCs-A (ASCs-T: healthy *n* = 8 and lipedema *n* = 10; ASCs-A: healthy *n* = 9 and lipedema *n* = 8). Results are displayed as scatter plots with bar. Values are mean ± SEM. *$p < 0.05$, **$p < 0.01$.

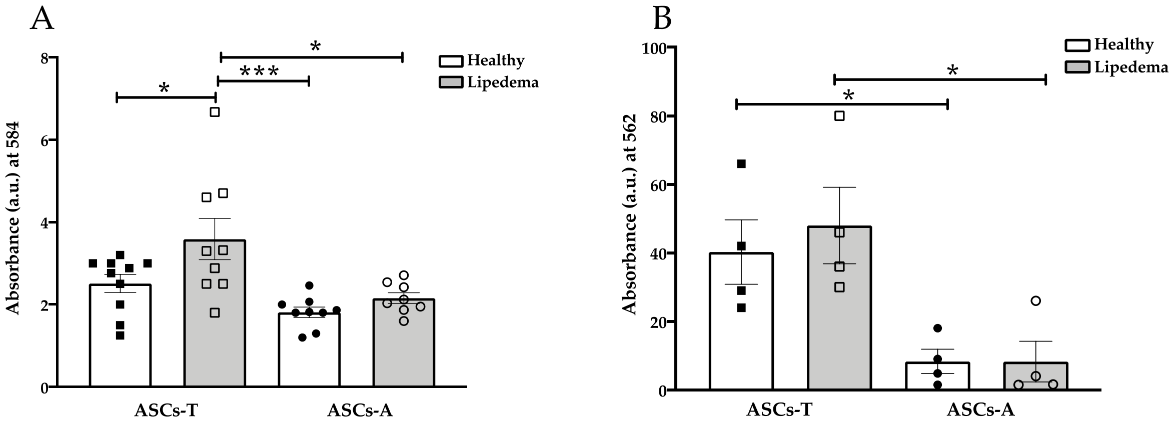

Figure S4. Quantitative analysis of Oil Red O (A) and Alizarin red (B) by spectrophotometry showing a significant decrease in adipogenic and osteogenic differentiation potential in lipedema ASCs-A compared to lipedema ASCs-T ($p < 0.05$) and a significant decrease in the osteogenic potential in healthy ASCs-A compared to healthy ASCs-T, but no difference was shown in their adipogenic potential (Adipogenic differentiation ASCs-T: healthy *n* = 10 and lipedema *n* = 9, ASCs-A: *n* = 8 per group, Osteogenic differentiation, *n* = 4 per group).
